# Prediction of Asthma Risk Using Family Health Histories identified from Population-based Electronic Healthcare Records.

**DOI:** 10.23889/ijpds.v7i3.1952

**Published:** 2022-08-25

**Authors:** Amani Hamad, Lin Yan, Joseph Delaney, Mohammad Jafari Jozani, Pingzhao Hu, Shantanu Banerji, Lisa Lix

**Affiliations:** 1 University of Manitoba; 2 CancerCare Manitoba

## Objectives

Prediction of asthma risk can potentially be improved by including family history of asthma and related diagnoses, which reflect both genetics and shared environments. We tested the improvements in offspring asthma risk prediction using objectively-measured maternal, paternal, and offspring histories of comorbid conditions from administrative healthcare databases.

## Approach

A population-based cohort study was conducted using data from Manitoba, Canada. Children born from 1974 to 2000 with linkages to at least one parent using family identification numbers were included. Asthma diagnosis and comorbidities were identified from hospital and outpatient physician visit records. Lasso regression models were used to assess performance and identify important predictors. The base model included offspring demographics, diagnosed allergic conditions and respiratory infections, and diagnosed parental asthma. Subsequent models included multiple comorbid chronic health conditions for offspring and parents.

## Results

The cohort included 195,666 offspring; 51% were males, 13.6% had a parental asthma diagnosis, and 17.7% had an asthma diagnosis (median age at diagnosis: 6.0 years; interquartile range 3.0-11.0 years). The base model achieved a modest prediction performance with an area under the receiver operating characteristic curve of 0.60, sensitivity of 0.46 and a specificity of 0.67 using a threshold of 0.20. Sensitivity significantly improved when we included offspring chronic health conditions (sensitivity= 0.69; specificity = 0.66); both measures further improved when we additionally included parents’ chronic health conditions (sensitivity= 0.72; specificity = 0.70). Chronic obstructive pulmonary disease, noninfectious gastroenteritis and otitis media were among the variables that added incremental predictive value of asthma risk with odd ratios of 1.36, 1.25 and 1.18, respectively.

## Conclusion

Including offspring and parents’ chronic health conditions, identified objectively from administrative healthcare databases, improved the performance of asthma risk prediction models in children. Health histories of comorbid conditions provide important factors to improve risk prediction models of chronic health conditions, which will facilitate disease prevention and treatment strategies.

